# Clinical neuropathology practice guide 5-2013: markers of neuronal maturation 

**DOI:** 10.5414/NP300638

**Published:** 2013-07-25

**Authors:** Harvey B. Sarnat

**Affiliations:** Departments of Paediatrics, Pathology (Neuropathology) and Clinical Neurosciences, University of Calgary Faculty of Medicine and Alberta Children’s Hospital Research Institute, Calgary, Alberta, Canada

**Keywords:** cellular maturation, neuroblast, neuronal markers, NeuN, synaptophysin, chromogranins, enolases, microtubule-associated proteins, calretinin, calbindin, acridine orange, silver impregnations, luxol fast blue, mitochondrial enzyme histochemistry

## Abstract

This review surveys immunocytochemical and histochemical markers of neuronal lineage for application to tissue sections of fetal and neonatal brain. They determine maturation of individual nerve cells as the tissue progresses to mature architecture. From a developmental perspective, neuronal markers are all about timing. These diverse cellular labels may be classified in two ways: 1) time of onset of expression (early; intermediate; late); 2) labeling of subcellular structures or metabolic functions (nucleoproteins; synaptic vesicle proteins; enolases; cytoskeletal elements; calcium-binding; nucleic acids; mitochondria). Apart from these positive markers of maturation, other negative markers are expressed in primitive neuroepithelial cells and early stages of neuroblast maturation, but no longer are demonstrated after initial stages of maturation. These examinations are relevant for studies of normal neuroembryology at the cellular level. In fetal and perinatal neuropathology they provide control criteria for application to malformations of the brain, inborn metabolic disorders and acquired fetal insults in which neuroblastic maturation may be altered. Disorders, in which cells differentiate abnormally, as in tuberous sclerosis and hemimegalencephaly, pose another yet aspect of mixed cellular lineage. The measurement in living patients, especially neonates, of serum and CSF levels of enolases, chromogranins and S-100 proteins as biomarkers of brain damage may potentially be correlated with their corresponding tissue markers at autopsy in infants who do not survive. The neuropathological markers here described can be performed in ordinary hospital laboratories, not just research facilities, and offer another dimension of diagnostic precision in interpreting abnormally developed fetal and postnatal brains.

## Introduction 

The labeling of both normal and abnormal cellular expression of proteins and metabolic products in tissue sections for light microscopy has enabled more precise and objective histopathological diagnosis of many neurodegenerative diseases and neural neoplasms. The various immunocytochemical and histochemical markers are equally useful in the context of ontogenesis, but have received relatively less attention. Documentation of normal development is primordial to an understanding of malformations, including effects of genetic mutations and epigenetic prenatal insults on the developing nervous system at various stages of maturation. 

Markers of cellular maturation in tissue sections are immunocytochemical reactivities or histochemical stains employed to denote a stage of differentiation or cellular maturity. Positive markers of differentiation are based upon the expression of proteins not present until a degree of maturation is achieved. These comprise the majority. Negative markers are those of immaturity which label transitory proteins present only in undifferentiated cells or during early stages of differentiation, usually then regressing as maturation proceeds or substituted by more mature neuronal proteins serving a similar function. 

The various positive markers of neuronal maturation may be divided into those that are expressed early, intermediate or late in neuronal differentiation (Table 1, Table 2). Early markers are defined by neuroepithelial expression before cellular migration, as well as during the migratory journey and after cells reach their destination. Intermediate markers are not expressed in neuroepithelium, but appear during the migratory course and persist. Late markers are not expressed in undifferentiated neuroepithelial cells or during migration, and often not even after neuroblasts reach their final mature position in the brain, but only after acquiring functions as mature neurones, including biosynthesis of neurotransmitters, resting membrane potential, membrane receptors and synaptic relations. Examples of early markers are microtubule-associated protein-2 (MAP2) and calretinin. Examples of late markers are NeuN and synaptophysin. 

An example of a negative marker in cells of neuronal lineage is non-neuronal enolase in early differentiation, replaced by neurone-specific enolase as the neuroblast matures [1]. Another example is the early and transitory expression of the intermediate filament protein vimentin in neuroepithelial cells and early differentiating neuroblasts. Amongst glial cells, recently proliferated and immature astrocytes express vimentin, but it is then replaced by the more mature intermediate filament protein glial fibrillary acidic protein (GFAP) of mature astrocytes. [2]. Both vimentin and GFAP are transitory proteins in fetal ependymal cells, neither of which has normal expression in mature ependymocytes [3]. Vimentin also is expressed in specialized neural progenitor cells, such as the radial glial cell, that retain a multi-potential capability of differentiating into many kinds of cells for regeneration in the presence of adverse epigenetic events, such as vascular or traumatic injury of the fetal brain. Immunocytochemistry offers an objective means to document lineage and differentiation of neuronal progenitor stem cells in tissue sections [4] that are available to the practicing neuropathologist. 

An understanding of pathological maturation and malformations of the fetal brain cannot be achieved without primary control standards of normal development. Genetic defects in programming of brain development affect not only morphogenesis, but also the timing of onset of the various developmental processes, which can be either delayed or precocious. Furthermore, malformations must be considered not only in the context of tissue architecture or arrangements of cells and their processes, but also the temporal relations of simultaneous expression of different developmental processes. This temporal feature includes both the earliest expression and the length of the embryonic or fetal period over which the abnormal genetic effect is active. 

The historical context of the discovery and application of each of the markers now used is preserved in this review by citation of the early works as well as the most recent advances. Many gaps in our knowledge of the ontogenesis of these markers remain to be filled. There also exists a large literature on the use of these markers in cell cultures and in tissue sections of neural neoplasms, but these topics are beyond the scope of the present review. 

## Immunocytochemical markers 

These immunoreactivities are all applicable to formalin-fixed, paraffin-embedded sections, but some are more resistant to post-mortem autolysis than others. Some are nuclear and others, cytoplasmic, markers. Examples of nuclear markers include neuronal nuclear antigen (NeuN), Ki67 or MIB-1 proliferating cell nuclear antigen, those of DNA viruses such as Herpes and neural tumour markers such as p53 and isocitric dehydrogenase (IDH-1 and IDH-2 mutations). Nuclear labeling is an advantage over cytoplasmic markers in studying fetal brain because immature nerve cells usually have sparse cytoplasm but large, vesicular nuclei, so labeling is easier to detect. It also more easily enables double labeling with another marker localized to the cytoplasm. A disadvantage of nuclear labels are that in general nuclear antigens are more rapidly degraded post-mortem, so that reactivity is no longer reliable after 24 hours of autolysis. 

### Nucleoprotein markers 

#### Neuronal nuclear antigen (NeuN)

NeuN is a low molecular weight (46 – 48 kDa) nucleoprotein, originally designated *mAb A60* [5, 6]. It is recognized by a specific antibody and may be demonstrated immunocytochemically in most, but not all, neurones of the central and peripheral nervous system in humans and other vertebrates, but not in invertebrate nerve cells [5, 6, 7]. NeuN is now identified as *Fox3*, a new member of the *Fox1* gene family of regulatory splicing factors [8, 9]. Glial and ependymal cells are non-reactive for NeuN. 

Specific neurones never recognized by NeuN antibody are: Purkinje cells, dentate and inferior olivary neurones of the cerebellar system, though granule cells and pontine nuclei are reactive; photoreceptor cells of the retina; mitral cells of the olfactory bulb; sympathetic chain ganglion cells, though adrenal medullary chromaffin cells and parasympathetic neurones of the myenteric plexi are immunoreactive; Cajal-Retzius cells of the molecular zone of the fetal cerebral cortex. Those neurones that are non-reactive in adult life [5, 6] also never exhibit transitory reactivity during development [7, 10]. They share no functions, fibre connections or neurotransmitters in common, hence the reason for their lack of NeuN nucleoprotein was not evident. The explanation may lie, however, in the negative regulation of gene expression. Many neurone-specific markers are programmed by what molecular geneticists call “pan-neuronal genes”. These genes are regulated by neural-restrictive silencer elements (NRSE) which are bound by neurone-specific silencer factor (NRSF), also known as REST. REST is a suppressor, so that if it is expressed, pan-neuronal genes are not expressed. REST thus keeps pan-neuronal genes turned off in non-neuronal cells and also in certain classes of neurones that express REST [11, 12, 13]. Furthermore, the proneural gene *Neurogenin-2* (*Ngn2*) is expressed through a basic helix-loop-helix (bHLH) transcription factor in early neuronal progenitor cells but only specifies early differentiating deep-layer pyramidal neurones arriving at the cortical plate exclusively by radial migration. *Ngn2* is temporally regulated by GSK3 kinase and declines in expression during corticogenesis with progressive phosphorylation of GSK3 [14, 15, 16]. GSK3 kinase thus probably regulates the expression not only of *Ngn2* but also of NeuN. 

**Figure 1:**
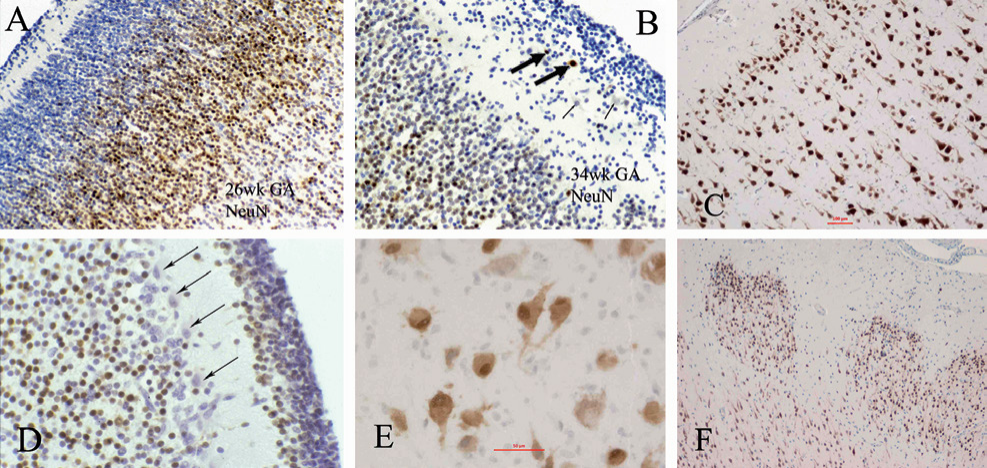
Figure 1. Neuronal nuclear antigen (NeuN) immunoreactivity in (A, B) cerebral cortex shows progressive neuronal maturation from deep to middle layers: A: At 26 weeks gestation, nuclear reactivity is seen only in the deep layers of cortex and in white matter neurones just beneath the cortical plate. B: At 34 weeks gestation, most neurones of Layer 2 are still not yet mature enough to exhibit NeuN-reactive nuclei, but most of those in all other layers are reactive. By term, neurones in all layers are reactive (not shown). In B, Cajal-Retzius (CR) neurones (thin arrows) normally are non-reactive at all ages, whereas reactive nuclei in the molecular zone (thick arrows) identify over-migrated non-CR neurones. C: The cytoplasmic epitope of NeuN appears during post-natal development, as seen in this frontal neocortex of a 3-year-old girl; the nuclear reactivity continues to be strongly expressed. Cytoplasmic NeuN extends into the proximal dendritic trunk, though not the axone, thus showing orientation of the neurone. D: Cerebellar cortex of a 26-week fetus. NeuN shows nuclear labeling in half of the internal granule cells, as well as in the deep lamina of the external granular layer; pre-migratory granule cells already have extended axones as longitudinal fibers in the molecular zone, forming synapses with Purkinje cell dendrites, hence are mature neurones before migration. Purkinje cells (arrows) are not labelled at any age. E: Megalocytic and dysplastic neurones in a surgical resection of focal cortical dysplasia Type IIb of a 4-month-old boy shows NeuN reactivity as both nuclear and cytoplasmic epitopes in mature neurones, even if the cells are dysplastic. F: Nodular architecture of the paramedian frontal neocortex of this 25-week fetus with holoprosencephaly shows haphazard lamination, but the majority of NeuN-reactive neurones are near to the surface of the nodules, not deep at the base, showing an inversion of the migratory pattern; in addition the number of reactive neurones in the cortex is greater than expected at this age, indicating precocious maturation. Many heterotopic deep white matter neurones also are reactive. (A, B, D, F) × 100, original magnifications.

Amongst cytologically abnormal cells in focal cortical dysplasia Type 2b and in certain other malformations, balloon cells and others of mixed lineage are non-reactive for NeuN [17]. Megalocytic and dysplastic neurones in these same malformations, by contrast, generally do express nuclear NeuN (Figure 1E). Inter-specific differences may occur, limiting extrapolation to humans of animal models. For example, dopaminergic neurones of the substantia nigra are highly variable in their NeuN reactivity in rodents [18, 19], but are consistently reactive in human substantia nigra. The detection of NeuN in paraffin sections by conventional light microscopy using diaminobenzine tetrahydrochloride chromagen is superior to immunofluorescent labeling [20]; a protocol also is available for its demonstration by confocal microscopy [21]. 

NeuN is a particularly valuable marker because of its late expression in neuronal maturation, generally not before reactive neurones are functional secretory cells. Its nuclear reactivity enables detection when somatic cytoplasm is still sparse. Later in cytological maturation, a cytoplasmic epitope also appears and also shows the dendritic trunk to denote orientation of the neurone, but it is the nuclear reactivity that remains more important as a maturational marker with a consistent and predictable ontogenetic pattern. For example, neuroblasts are not reactive during the course of radial migration to the cerebral cortex, though some become reactive as they approach the cortical plate and most only become reactive after achieving their final position within the cortex [7]. Reactivity in cortical neurones appears first in the deepest layers at 22-week gestation; by 34 weeks it is present in the superficial layers as well (Figure 1A, B), though scattered nonreactive cells persist in Layers 2 and 3 at term. Postnatally, a cytoplasmic epitope appears in the neuronal soma and proximal dendritic trunk, showing orientation of the neuronal poles, but nuclear reactivity remains most important as a marker of maturation (Figure 1C). In some malformations, NeuN can detect not only an inversion of the cortical layering but precocious maturation of the neurones; this feature is particularly evident in holoprosencephaly (Figure 1F). In the hippocampus, both granule cells of the dentate gyrus and pyramidal cells of Ammon’s horn are labeled by NeuN as they mature. 

NeuN reactivity never occurs in sympathetic ganglion cells in the peripheral autonomic nervous system, but does occur in parasympathetic neurones of the submucosal and myenteric plexi [22] and may occur surprisingly as early as 9 weeks in the enteric tract [Yu W, Pinto-Rojas A, Sarnat HB, in preparation]. During secondary neurulation, some immature cells of the conus medullaris express NeuN (and also synaptophysin) precociously, which at that early stage of development may play a different role in modulating the differentiation of structures than their later function in neuronal maturation and synaptogenesis [23]. The paradox of early expression of NeuN in rare scattered neuroblasts in the telencephalic periventricular germinal matrix [7] may be another example, at the opposite end of the neural tube. Müller et al. [5] found that immunocytochemically detectable NeuN protein first appears at the time of withdrawal of the primordial neuroblast from the cell cycle or with the initiation of terminal differentiation of the neurone, the two extremes of neuronogenesis. 

In the cerebellar cortex, some pre-migratory cells of the deep half of the external granular layer are reactive at mid-to-late gestation, but near term these external granule cells seem to lose their reactivity; internal granular cells become slowly but progressively reactive (Figure 1D) [7, 8]. The NeuN reactivity of pre-migratory external granule cells is probably because they are already more mature than pre-migratory neuroblasts of the cerebral cortex; in the cerebellum they already have extended their axones as parallel fibers in the molecular zone and established synaptic contacts with Purkinje cell dendrites before migrating. Why the later migratory cells of the external granular layer and some internal granule cells remain non-reactive until later is not fully explained. 

Post-mortem degradation of the NeuN nucleoprotein is rapid, hence it is not a reliable developmental marker if more than 24 hours has transpired between death and tissue fixation. If the fetus or neonate has suffered an hypoxic/ischaemic injury, however, loss of NeuN reactivity does not necessarily indicate neuronal death and may be a transitory impairment, at least in rodents [24]. Ischaemic preconditioning, by contrast, enhances precocious neuronal maturation in rats [25]. Caution, therefore, must be exercised for NeuN interpretation in the perinatal period in humans. NeuN reactivity in prepared slides does not fade with time. 

### Synapatic vesicle markers 

#### Synaptophysin

This molecule, first identified in 1985, is a 38 kDA glycoprotein comprising one of the principal structural elements of the walls of synaptic vesicles [26, 27]. It is non-specific in respect of the type of neurotransmitter contained by the vesicle, site of the synapse (axosomatic or axodendritic) or function (excitatory or inhibitory). Synaptophysin reactivity is seen at the neuronal membranes when synapses form, thus it is a late marker of neuronal maturation. In fetal white matter, reactivity often is seen within immature, but not mature, axones. The likely reason is that the synaptophysin protein is synthesized in the somatic cytoplasm and transported in the axone to the terminal, where it is combined with other proteins, such as synaptotagmin and synaptobrevin, in the assembly of the synaptic vesicular walls; during axoplasmic flow, the molecule is complete enough that it is recognized by the antibody, as noted in the original descriptions of the immunocytochemical antibody [26, 27, 28]. The synaptophysin molecule has been purified and characterized for its ion channel activity [29]. Endocytosis of synaptophysin requires the carboxy-terminus of the molecule [30]. Unlike NeuN, synaptophysin is a very robust molecule that resists autolytic degradation for as long as 5 days post-mortem before fixation [31]. It is, therefore, very reliable for application to autopsy tissues. It also is reliable for new sections cut from paraffin blocks as old as 25 years, providing a valuable tool for retrospective studies. 

**Figure 2:**
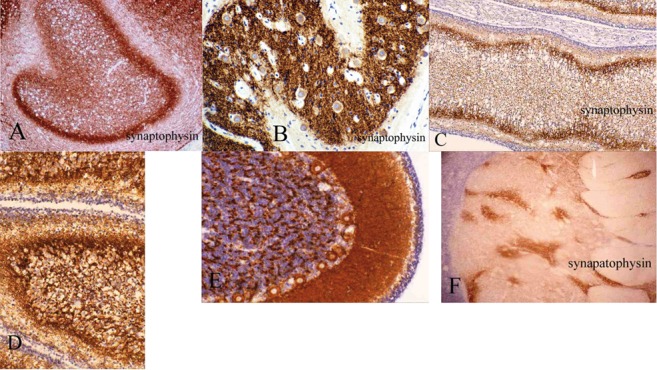
Figure 2. Synaptophysin reactivity in the inferior olivary nucleus at (A), 15 weeks, and (B) 33 weeks gestation. Reactivity at 15 weeks is limited to the periphery of the nucleus and is more intense medially, including the medial accessory olive, than laterally, following a medio-lateral gradient in the horizontal axis. By 33 weeks, reactivity is uniform throughout all parts of the nucleus and the crenations are now well demonstrated as well. Note also the perinuclear cytoplasmic reactivity in many neurones, indicating active synthesis of synaptophysin molecules before axonal transport. This also is seen in large sensory neurones of the dorsal root ganglia (not shown), where there are no synapses. C, D, E: The cerebellar cortex at (C) 15 weeks, (D) 28 weeks and (E) 40 weeks shows progressive synaptophysin reactivity beginning around Purkinje neurones. The vermis is ~ 2 weeks earlier in synaptogenesis than the lateral hemispheres until ~ 32 weeks, after which time no difference is perceived. F: Head of the caudate nucleus, adjacent to the anterior limb of the internal capsule, shows transitory patchy reactivity at 22 weeks that becomes uniform within the grey matter before term. This patchy pattern is seen throughout the corpus striatum, but not in the globus pallidus; it also occurs in the pontine nuclei of the basis pontis (not shown). (A) × 250; (B) 200 ×; (C, D, E, F) ×100, original magnifications.

Synaptogenesis in the brain is an ontogenetic process with precise spatial and temporal distribution. Immunocytochemistry is a practical and reliable method of inferring synaptogenesis by the sequence of appearance of reactivity in the cerebral cortex and hippocampus [31], corpus striatum, globus pallidus and substantia nigra [32, 33], cerebellar system [34, 35] and olfactory bulb [Sarnat HB, in preparation]. Not only does it denote the appearance and progression of synapse formation, but it also is valuable in fetal neuroanatomy in demonstrating with striking contrast the formation of the shapes of nuclei, such as the inferior olivary (Figure 2A, B) and dentate nuclei of the cerebellum better than hematoxylin-eosin or other histological stains. Synaptophysin also enables recognition in tissue sections of the gradients of genetic expression in each of the three axes of the neural tube, as these nuclei take form [34]. The periphery of these nuclei exhibits reactivity sooner than its central portions. The cerebellar cortex also exhibits a predictable pattern of synaptogenesis (Figures 2C, D, E) [35]. The synaptic sequence in the developing red nucleus has been studied [34, 36]. Within the corpus striatum (Figure 2F) [32, 33] and pontine nuclei in the basis pontis [35], a patchy distribution of synaptogenesis amongst subpopulations of neurones that histologically appear indistinguishable. Quantitation of synaptophysin reactivity in paraffin sections is now feasible [37] and quantitation of post-synaptic density proteomes in rat forebrain and cerebellum also has been demonstrated [38]. 

Immunocytochemical antibodies against the synaptic vesicle wall proteins synaptotagmin, synaptobrevin and SNAP-25 also are available, but data are more limited to denote their fetal expression and similarity to synapatophysin. In human neuropathology, synaptophysin has been widely used in the diagnosis of neural tumours and neurodegenerative diseases, but its application as a maturational marker has been more limited until recently [28, 29, 30, 31, 32, 33, 34, 35, 36]. Synapsin-1 and -2 are associated with synaptic vesicles, but are not molecular structural elements of the vesicular wall. A unique pre-synaptic extracellular protein matrix of around functional synapses in the hippocampus is demonstrated in adults with Alzheimer disease, but does not normally occur in most mammals including humans [39]. 

The autosomal gene *SYNGAP1* encodes a protein critical for synaptic function. Disruption of this gene is a well-documented cause of non-syndromic autosomal dominant intellectual deficit and autism [40]. A result of *Syngap1* mutations or deletions in the mouse is impairment of the formation and maturation of dendritic spines during development, particularly those associated with NMDA-type glutamate receptors [41, 42]. An immunocytochemical antibody for the transcription product of this gene is under development and will provide a correlate for synaptophysin reactivity. 

#### α-synuclein 

This small 140 amino acid protein is localized in pre-synaptic axonal terminals. Overexpression of a phosphorylated mutant is characteristic in several neurodegenerative diseases, and comprises the major component of Lewy bodies; fibril formation and protein aggregation as a principal pathogenetic mechanism [43, 44, 45]. Fibrils of α-synuclein form unique neurone-to-neurone non-synaptic transmission by axosomatic extension [46] and it is particularly neurotoxic for dopaminergic neurones [47]. Glial cell dysfunction also occurs in the α-synucleinopathies [48]. For purposes of demonstrating synaptogenesis in the human fetal brain, α-synuclein is not presently useful. Even in neurodegenerative diseases, overexpression of α-synuclein was found to be reliable with inter-observer consistency in a European consortium of neuroxpathologists, only in the cerebral cortex [49]. This inconsistency may be related in part to a variety of immunocytochemical techniques for demonstrating it [50, 51], which also might yield variable results in developing brain. 

The α-synuclein: synaptophysin messenger RNA and protein ratio was high during early development in rats but low in the adult, suggesting a higher expression of α-synuclein per synapse during early synaptic development [52]. A systematic study of this presynaptic protein in immature human brain was provided by Raghavan et al. [53]. They observed perisomatic expression in the neocortical plate as early as 11 weeks gestation, long before synapse formation; by 20 weeks several discrete neuronal groups were reactive for α-synuclein in the hippocampus, basal ganglia, and brainstem tegmentum, which persisted for the first few years. Cerebellar α-synuclein is first detected at 21 weeks gestation, in the internal granular and molecular layers, but not in relation to Purkinje cells or dentate nuclear neurones; cerebellar reactivity persists into adult life [53]. 

#### Chromogranins 

These small, glycosylated, calcium-binding, acidic proteins are widely distributed in neurones, chromaffin and other neuroendocrine cells. They are ultrastructurally localized to secretory granules and synaptic vesicular walls [54, 55, 56, 57, 58, 59, 60], but differ from other vesicular wall proteins such as synaptophysin because chromogranins are water-soluble, hence are secretory rather than structural proteins. 

Three principal chromogranins, designated *A, B* (formerly *secretogranin I*) and *C* (formerly *secretogranin II*), comprise a family with different molecular weights amongst various mammals. Chromogranin-A (CgrA), molecular weight 68 – 75 kDa, is the most studied and is widely distributed in the human brain; its molecular structure and the gene regulating it were described in 1987 by Gratzl [59]. The neuroanatomical distribution and release of chromogranins must be considered in relation to the specific neurotransmitters with which they are associated, such as acetylcholine, the monoamines, GABA and neuropeptides, as well as the post-synaptic membrane receptors for these transmitters [57, 61, 62, 63]. Whereas chromogranins themselves are not transmitters, they serve to modulate the release of transmitters and thus help to regulate neuronal excitability. Chromogranins were originally described in the adrenal medulla and closely associated with the release of epinephrine [57, 59, 64, 65, 66, 67, 68]. 

**Figure 3:**
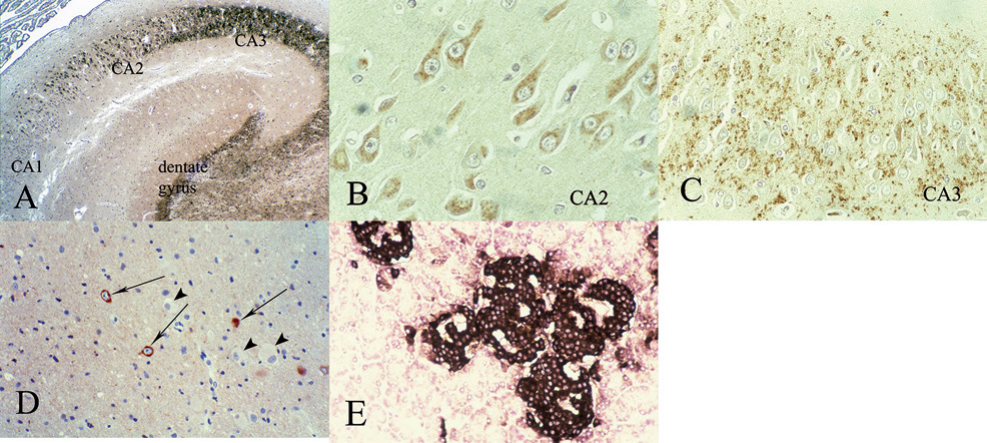
Figure 3. A: Chromogranin-A (CgrA) reactivity in the normal hippocampus of a neonate of 38-weeks gestation. Strong reactivity is seen in the granule cells of the dentate gyrus and in the neuronal cytoplasm, but not the neuropil, of the CA2 sector of Ammon’s horn. In the CA3 sector, cytoplasmic reactivity in the somata is less intense than in CA2 and the neuropil shows many beaded processes; these are axones of the neurones in CA2 that project to CA3 and to CA1 without reciprocal connections back to CA2. CA1 is the last sector of Ammon’s horn to mature, in postnatal life, and its neurones do not yet exhibit much CgrA at near-term. B, C: CgrA reactivity in normal Ammon’s horn of a 2-month-old infant shows (B) granular reactivity in the somata, but not the neuropil of the CA2 sector; (C) in CA3, sparse or no reactivity is seen in neuronal somata, but beaded axonal reactivity is found throughout the neuropil, within terminal axones of CA2 neurones. D: Surgical resection for epilepsy of a zone of focal cortical dysplasia Type 2a of a 3-year-old boy; normal-appearing neurones show strong CgrA reactivity (arrows), whereas dysplastic, megalocytic neurones exhibit no CgrA (arrowheads), unlike NeuN. E: CgrA reactivity is strong in islet cells, but not in exocrine cells of pancreas. CgrA is a neuroendocrine cell marker and serves as a good control for brain. (A) × 40; (B, C, D) × 200; (E) × 250, original magnifications.

Cytological localization of chromogranins is in the neuronal soma with axonal transport to terminals where they form granules and contribute to the walls of synaptic vesicles, in both the central and peripheral nervous systems [64, 65]. CgrA occurs in axones of all sizes, but CgrB is limited to large axones. CgrB is most closely associated with cholinergic neurones, including spinal motor neurones; CgrA is demonstrated in both cholinergic and adrenergic neurones [65]. CgrA also distinguishes monodendritic neurones of the cerebral cortex [61] and inhibits dopamine release in the retina [68]. Chromogranins in general are strongly expressed in monoamine-secreting neurones. They usually do not label dysplastic neurones in focal cortical dysplasias Type 2 (Figure 3D). 

During fetal development, neither CgrA nor CgrB are expressed in immature neurones despite a wide but specific distribution in mature neurones in the rat brain [64, 65, 66] and inhibits retinal dopamine release [68]. CgrA is of particular value in studying the maturation of the hippocampus, where the neuronal somata but not the neuropil of the CA2 sector of Ammon horn are labeled for CgrA, and the CA1 and CA3 sectors to which CA2 projects without reciprocity exhibit weaker somatic CgrA but strong reactivity in the neuropil, representing the terminal axones of CA2 (Figure 3A, B, C). Preliminary data indicate that this pattern is first observed at 18 weeks gestation [Sarnat HB, unpublished]. CgrA and CgrB are strongly over-expressed in the hypertrophic axones in aganglionic segments of bowel in infants with Hirschsprung disease [69]. CgrA may be a better marker of intestinal ganglion cells than synaptophysin, though the two may be complimentary if studied together [70]. Documentation of the ontogenesis of the chromogranins in the human fetal nervous system is incomplete. CgrA is a neuroendocrine cell marker, hence pancreas tissue can be used as a control because of the intense reactivity of islet cells but not of exocrine cells (Figure 3E). It also is a good marker of neuroendocrine tumour cells [71, 72]. 

### Glycolytic enzyme markers 

#### Neurone-specific enolase (NSE) 

Enolases are glycolytic enzymes found in many cells of the body, including fibroblasts. They catalyze the interconversion between 2-phophoglyceric acid and phophoenolypyruvate. Discovered in 1975 [73], two principal isozymes are now identified in the CNS: *neurone-specific enolase* (NSE; α-enolase) is synthesized by mature neurones and *non-neuronal enolase* (NNE; γ-enolase) is produced by glial cells and immature neurones [1, 74, 75, 76, 77, 78, 79]. NNE identifies astrocytes, including Bergmann glia, in the cerebellum [80]. 

Cytoplasmic reactivity of NSE is demonstrated in all types of neurones. It is an intermediate marker of maturation, not showing reactivity of cells still in the germinal matrix, but late in the course of neuroblasts migration and in the cortical plate. Strong background reactivity in the neuropil makes contrast poorer than with some other markers. This background reactivity is not artifactual, but rather is due to equally strong label in neurites as in the neuronal somata. Despite this disadvantage, NSE retains an important role as a maturational marker for two reasons. First, linked with M17Z as a construct in transgenic mice, it is an inhibitor of caspase-1-induced neural cell apoptosis [78]. This property has received attention as a potential therapeutic approach to several neurodegenerative diseases of human, including Huntington chorea and spinal muscular atrophy. 

A second reason that NSE immunoreactivity in tissue sections of brain remains important is that clinicians, including neonatologists, increasingly measure NSE and NNE levels in serum and especially in cerebrospinal fluid (CSF) as a biomarker of “brain damage” in premature and term neonates, as well as older children, who have suffered hypoxic-ischaemic and other encephalopathies [81, 80, 82, 83, 84]. Serum NSE remains elevated in children after a clinical diagnosis of brain death [85]. In infants who do not survive, it may be useful to correlate neuronal NSE in tissue sections of cortex and other structures with elevated pre-mortem CSF concentrations. Elevated serum and CSF levels also have been reported status epilepticus and other epileptic conditions [86, 87, 90, 89, 90, 91, 92]. It is not a totally reliable biomarker because some patients, in whom high levels are anticipated clinically, do not exhibit elevations in CSF enolase [75]. Furthermore, the serum NSE level in 42 children with uncomplicated epilepsy was not a sensitive or reliable biomarker of brain damage or dysfunction [93]. Ventricular tapping does not influence serum concentrations of NSE [94]. Control values of NSE in the CSF of neurologically healthy children have been documented [95]. At present the clinico-pathological correlations remain investigative rather than a practical tool in diagnostic neuropathology. 

**Figure 4:**
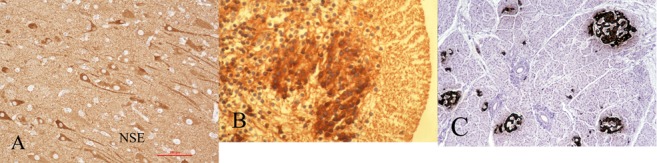
Figure 4. Neurone-specific enolase (NSE) reactivity in (A) a surgical resections of the right temporal neocortex of a 16-month-old boy with a highly epileptogenic mild focal cortical dysplasia Type 1 at that site. Background reactivity of the neuropil is not as intense as the soma, but in some cases is so strong that it obscures the contrast of the neuronal soma, limiting its value as a marker. B: NSE is strongly expressed in spinal motor neurones of the ventral horn of a 10-week fetus; innervation of striated muscle occurs at 9 – 11 weeks. C: Pancreatic tissue of a term neonate serves as a control because of the selective intense NSE reactivity of insulin-secreting beta cells of the islets of Langerhans, similar to other neuroendocrine cells; pancreatic exocrine cells are non-reactive. (A) × 100; (B) × 250; (C) × 200, original magnifications.

Immunocytochemical studies of CNS enolases during the course of fetal development demonstrate that maturing neuroblasts switch from NNE to NSE production after completing migration to the cerebral cortex, in the cerebellum and in brainstem nuclei, in the rat [1, 80, 96], monkey [1] and in the human [84]. Measurement of each isozyme by radioimmunoassay during development demonstrates that NSE levels are low in embryonic rat brain and increase at a time corresponding to morphological and functional maturation of neurones; NNE levels are high in embryonic brain and decrease when NSE first appears, followed by a gradual increase to adult levels [78]. These enolases thus provide useful markers of neuroblast maturation in sections of human fetal, neonatal and post-natal brain (Figure 4A). 

Neurones of the peripheral nervous system including chromaffin and other neuroendocrine cells such as the insulin-secreting islet cells of the pancreas also show NSE synthesis (Figure 4C) and a similar maturational pattern [97]. Pancreatic beta cells also can serve as controls for chromogranins and S-100β protein. 

The most detailed regional immunocytochemical study of NSE in the human is that of Nishimura et al. in 1985 [98]. They identified NSE-containing neurones in 17 week fetuses in the facial and cochlear cranial nuclei, in neurones of the brainstem reticular formation and in about half the neurones of the globus pallidus and the thalamus; centromedian thalamic nuclei showed stronger reactivity than did the lateral and medial groups, but by 28 weeks gestation all thalamic neurones displayed equally strong NSE reactivity. Putamenal neurones showed reactivity as well at 28 weeks, considerably later than those of the globus pallidus. In the cerebellum, they found no reactivity at 14 weeks, but at 21 weeks most Purkinje cells and the majority of dentate nucleus neurones were strongly immunoreactive; Golgi cells became reactive at 28 weeks; at 32 weeks neuronal processes of the molecular zone and internal granular layer were recognized by NSE antibodies, but by 40 weeks only occasional Purkinje cells still were intensely reactive. No NSE or NNE immunoreactivity is demonstrated in undifferentiated neuroepithelial cells in the cerebral periventricular germinal matrix (subventricular zone) or in the external granular layer of the cerebellar cortex in humans or rodents. Spinal motor neurones exhibit strong NSE as early as 10 weeks (Figure 4B), during the 9 – 11 weeks period of innervation of striated muscle. 

The developmental pattern of NSE in the human occipital lobe striate (primary visual) cortex first exhibits immunoreactivity in pyramidal neurones of Layer 5 at 21 weeks, followed by those of Layer 3 at 24 weeks, but the granule cells, including those of the prominent layer 4 in striate cortex, do not exhibit NSE reactivity until 34 weeks gestation [98]. The parietal (somatosensory) neocortex of the rat shows a correlation of NSE reactivity within the various laminae with the arrival of afferent axones and, presumably, synaptogenesis [99]. Data are lacking for the hippocampus. 

### Markers of cytoskeletal elements 

#### Microtubule-associated proteins-2 and 1/5 (MAP-2; MAP-1/5) 

**Figure 5:**
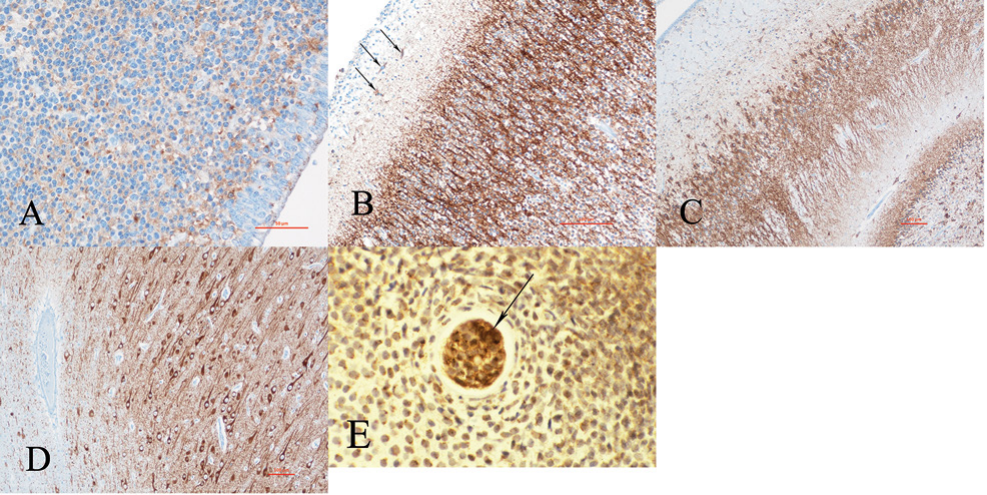
Figure 5. A, B, C: Microtubule-associated protein-2 (MAP2) immunoreactivity in a 32-week fetus, showing reactivity in all neurones (A) germinal matrix neuroepithelium shows weak reactivity in many immature neuroblasts; (B) temporal neocortex; Cajal-Retzius neurones (arrows) are intensely reactive, as are immature and mature neurones in all layers of the cortex; (C) hippocampus, including portion of dentate gyrus and CA2 sector of Ammon’s horn; D: Deep layer of frontal neocortex and adjacent white matter of a 3-year-old boy. E: reactivity in notochordal cells (arrow) and also in chondroblasts of a sclerotome forming the vertebral body in an 8-weeks fetus. (E) × 400, original magnification.

Microtubule-associated (MAP) proteins are a large family of cytoskeletal stiffening proteins that change in degree of phosphorylation with maturation of neural cells [100, 102, 103, 104, 105, 106]. Antibodies against MAP proteins exist in several forms that belong to the IgG1 subclass. They recognize neurones but not mature glial cells, ependyma or cells of mesodermal origin such as endothelial cells and microglia, in normal brain. Microtubules are structural proteins of the cytoskeleton and are expressed as early as the mitotic phase of cellular proliferation. They are involved in several fundamental developmental processes, including cellular motility, shape, polarity, remodeling of dendritic spines and intracellular transport and secretion and trafficking of cargo molecules to pre-, post- or extra-synaptic domains [104]. MAP proteins are not specific for neural cells. Many other cells of the body also have microtubules, including in the embryo and fetus, hence early immunocytochemical expression in these tissues as well. An example relevant to developmental neuropathologists is the demonstration of MAP proteins in the notochord and chondroblasts of the sclerotome during formation of the vertebral body (Figure 5E). 

Immunocytochemical antibodies against MAP proteins are in general use in diagnostic neuropathology as selective neuronal markers. MAP-2 antibody the most widely used; it labels the cytoplasm of the neuronal cell body and basal dendrites, but does not demonstrate the axone because the type of phosphorylation prevents MAP-2 from entering the axone by its N-terminal projection domain, creating a blockage at the axonal hillock (Figures 5B, D) [91, 92, 93]. MAP-1 (previously MAP-1A) and MAP-5 (previously MAP1B) are probably the same, hence now designated MAP1/5. This antibody, by contrast, is selective for the axone, and appears early with axonal outgrowth, helping establish neuronal polarity through changes in phosphorylation [103]. Five sub-groups of MAP-2 proteins are now identified as A, B, C, D and E. MAP-2E is unique in reacting exclusively in neuroglia rather than neurones [107], though MAP1/5 and MAP2 often react also in young oligodendrocytes during myelination [107]. Glial cells also require a cytoskeleton to maintain their shape and extend processes. 

MAP2 exists in high and low molecular weight isoforms, ranging from 70 to 280 kDa, MAP2A, 2B, 2C and 2D, and some tubulin-binding repeats are specific for some neurones, such as dorsal root ganglion cells and not for others; the ratio of subtypes also changes during maturation [102, 103, 104]. Isoforms of MAP2 have specificity in intra-neuronal distribution: MAP2B mRNA is mainly localized in dendrites and MAP2D is more concentrated in cell bodies of cultured fetal and adult rat hippocampal neurones [103]. 

MAP-2 is an early marker of neuronal maturation. Pre-migratory neuroepithelial cells show differentiation of neuronal lineage by exhibiting weak MAP-2 reactivity (Figure 5A), weak in part because of their sparse cytoplasm; reactivity increases in intensity during neuroblast migration and persists at maturity. MAP2 is helpful in identifying heterotopic neurones in the deep white matter and in studying neuronal density, orientation and architecture of the cerebral cortex. All neurones of the hippocampus (Figure 5C) and of the cerebellum (not shown) are reactive at all ages. 

Cajal-Retzius cells of the molecular zone of the fetal cerebral cortex, are strongly immunoreactive (Figure 5B), even early as 6 weeks gestation before the first wave of radial neuroblast migration. MAP2 reactivity was detected in human fetal spinal cord by Giovanini et al. [108] and in fetal cerebral cortex by Honig et al. [109] in the differentiating cortical plate at 14 weeks gestation, including Cajal-Retzius cells and subplate neurones. Chun and Shatz [110] demonstrated MAP2 immunoreactivity in the earliest generated neurones of the cerebral cortex of the fetal kitten. 

MAP-1/5 is earliest identified in neuroepithelial cells of the ganglionic eminence [103, 111]. It also becomes reactive during axonal maturation. Sensory neurones of the dorsal root ganglia express at least four MAP2 forms [112]. In the human fetal brain, its expression is characterized by axonal reactivity at 13 weeks gestation in the molecular layer of the cerebral cortex, with a rapid increase at 20 – 26 weeks, and in the sub-cortical white matter, increasing appreciably at 24 – 32; cortical pyramidal cells become increasingly reactive after 28 weeks and continue into adolescence [113]. In the cerebellar cortex, MAP reactivity also was detected at 13 weeks in the molecular zone and deep white matter, and showed detectable increase in the molecular layer between 36 weeks and 2 months post-natally and in the white matter at 20 – 22 weeks [114]. 

#### Class III β-tubulin (TUJ1) 

This microtubule-associated protein is useful because of its specificity in labeling only certain types of mature neurones, for example cerebellar Purkinje cells that are not recognized by NeuN [115]. This tubulin also is unique in being co-expressed with nestin and GFAP at 16 – 20 weeks gestation in both the periventricular zone and the neocortical plate, hence at mid-gestation this MAP protein is not yet specific for cells of neuronal lineage [116]. The early expression of this unphosphorylated MAP protein in primitive neuroepithelial cells indicates commitment to neuronal lineage [117]. Persistence of the co-expression of β-tubulin with GFAP or nestin in late gestation and post-natally denotes maturational delay or mixed lineage. A related tubulin-β-2 is expressed in early differentiating neuroepithelial cells and progenitor resident stem cells in human infant brain [118]. In the developing human adrenal medulla, there is a peak of expression during the active wave of migration of sympathetic neuroblasts, but in the mature medulla Class III β-tubulin is invariably present in adrenergic neurones, though not all chromaffin cells express it, hence its presence or absence may identify two subpopulations of these cells [119]. 

#### Doublecortin (*DCX*) 

The transcription product of this gene can be demonstrated immunocytochemically even in pre-migratory neuroblasts, hence is a good early marker of neuronal lineage in primitive neuroepithelial cells. *DCX* thus is a good early marker in human fetal brain and also in animal studies of neural cell lineage in neuroepithelium [120, 121]. Though a microtubule-associated protein, it regulates filamentous actin structure in developing neurones [122] and also regulates the microtubules of axonal growth cones [123]. In the dentate gyrus of the developing hippocampus, *Dcx* is expressed with initial dendritic growth, but diminishes before maturity [124]. It also promotes the migration of human progenitor cell-derived neurones in the dentate gyrus [125]. Another binding partner of *DCX*, apart from microtubules, is the cell adhesion molecule *neurofascin*, which localizes to the initial axonal segment in mature neurones and may be important in axonal guidance during development [126]. 

Because of the importance of DCX as a gene of neuroblast migration, its mutation or deficiency produces phenotypical expression as *subcortical laminar heterotopia* or *band heterotopia* [127]. This distinct disorder of neuroblast migration sometimes is incorrectly designated as *double cortex*, from which it derives its name, but the band or sheet of heterotopia in the deep white matter are not a true cortex by histological criteria. *DCX* is expressed early in neuroepithelial cells from their first expression of neuronal lineage and during migration, well before they mature as neurones [112]. The transcription product of the *ARX* and *LIS1* genes, other important facilitators of neuroblast migration and of organization of the cortical plate, also are microtubule-associated proteins that can be demonstrated immunocytochemically [127]. 

#### Tau-protein 

Tau is another microtubule-associated protein expressed early in neuroblast maturation as cytoskeletal elements, stabilizing microtubules in neurites and stiffening them [106]. Abnormal phosphorylated forms of tau are not expressed in any stage of normal brain development. Knockout mice deficient in tau-protein can survive, probably by partial compensation from other MAP proteins, but neuronogenesis and neuronal differentiation are delayed [128]. In addition to pathological phosphorylated forms of tau, abnormally acetylated tau or lack of acetylation, also may be associated with dementias [129, 130, 131]. Extracellular tau released by dying neurones may facilitate the spread of tauopathy to adjacent healthy neurones [128]. 

Once regarded as of little importance in developmental neuropathology, tauopathies were known only in adult neurodegenerative diseases of dementia, such as fronto-temporal lobar degeneration, Pick disease, Alzheimer disease and Parkinson disease with dementia [132, 133]. Infantile tauopathies now are well documented in focal cortical dysplasias Type 2 [134, 135], in which MAP-2 also is upregulated [138]. Tuberous sclerosis is another developmental malformation involving the mTOR activation pathway [137] in which tau is upregulated [Sarnat HB, unpublished observation]. Another malformation with prominent tauopathy in the perinatal period is hemimegalencephaly [138]. Tau-degradation is in part regulated by the *Akt* family of genes [139]; *AKT1* is mutated in Proteus syndrome, often with hemimegalencephaly, and *AKT3* is altered in non-Proteus HME. Tau expresses a different phenotype in the adult with dementia and neuronal degeneration than in the fetus or neonate with congenital malformation of the brain. During fetal development, the mutation of tau-protein or expression of abnormally phosphorylated forms results in abnormal cellular architecture and lineage, producing dysplastic and megalocytic neurones with abnormal neurites. Tau-proteins thus have acquired a new recognition as important developmental proteins and in future may become used with more frequency as markers of early neuroblast maturation. Up-regulation of abnormal phosphorylated tau-protein during early development can produce diseases classified as “infantile tauopathies”; they impair cellular differentiation by interfering with microtubular structure needed for the cytosketeton and formation of neurites. 

#### Growth-associated protein-43 (GAP-43) 

GAP-43 is another protein that has been applied as a marker of axonal growth, synaptogenesis and regeneration [140], but only limited data are available in the context of fetal development. GAP43 immunoreactivity is demonstrated throughout the neocortical plate, including axones of the molecular zone and subcortical white matter, from at least 14 weeks gestation, but not in undifferentiated neuroepithelial cells [109]. Cortical GAP-43 thus appears earlier than synaptic vesicles are demonstrated by synaptophysin immunoreactivity or by electron microscopy. It is strongly expressed in regenerating optic nerve in fish [141, 142], which may correlate with its high expression in growing fetal axones of the cortex and white matter. In kainic acid-induced seizures in rodents, GAP-43 also is seen in the cortex [143]. 

#### Neurofilament proteins (NFP) 

This family of intermediate filament proteins serve as cytoskeletal elements, together with microtubules and the more primitive intermediate filaments vimentin and internexin (see below), in the somatic and neuritic cytoplasm of both mature and immature nerve cells but not of glia. Three major subunits of NFP of 68 to 70, 150 to 160 and 200 kDa molecular weights, designated as NFL (light), NFM (medium) and NFH (heavy) [144]. All are available as immunocytochemical antibodies for application to brain tissue, as well as mixtures of 2 or 3 of the antibodies in the same incubating solution. They are synthesized in the soma and move by slow axonal transport [145, 146]. 

NFP proteins are specific for neurones but non-specific for the type of neurone; they differ during ontogenesis by the degree of phosphorylation, which changes with neuroblast maturation as the more highly phosphorylated forms are replaced by less phorphorylation [147]. Medium and heavy-chain NFPs can be used as markers of maturity in the developing fetal brain; their accumulation in axones not only serves as a convenient marker of axonal maturation, but promotes an increase in conduction velocity [147]. Immunoreactivity in auditory temporal cortex is first demonstrated at 22 weeks gestation and other cortical laminae express NFP after 1 – 12 years [148]. 

#### α-internexin 

Another ontogenetic change is the replacement of transitory primitive intermediate filament proteins by NFPs. Vimentin and nestin are examples of negative markers of neuroepithelial cells. Another is called *internexin*, which precedes and later coexists with NFP of low molecular weight in mature neurones; unlike vimentin and nestin, α-internexin is more specific for differentiating cells of neuronal lineage [149, 150]. α-internexin also is expressed in dysplastic neurones such as in tuberous sclerosis [151] and in gliomas [152]. The biochemical structure and chromosomal localization are known in the chicken [153] and human [152]. An intermediate protein called *peripherin* forms in axones of peripheral nerve as well as in the CNS and is closely related to internexin [144, 147]. It is an NFP for radial, as well as longitudinal, growth of the axoplasm, and in developing axones determines axonal diameter; phosphorylation is essential [147]. 

### Calcium-binding protein markers 

#### Calretinin, calbindin D28k and parvalbumin 

A family of proteins that bind calcium and serve not as neurotransmitters but modulators of neurotransmission have long been recognized as important indicators of neuronal maturation because levels of intracellular calcium ions are involved in regulating neurite outgrowth, growth cone motility in axonal pathfinding and expression of neurotransmitter receptors [154, 155]. Furthermore, calcium-binding proteins are involved in neuroblast migration because calcium ions regulate cell movement [156]. Calretinin and parvalbumin are also involved in neuroepithelial cell mitoses [157, 158]. Calcium is integral in other aspects of cellular maturation, including gene transcription and enzymatic activation [158, 159]. Some of these calcium-binding proteins are induced by vitamin D [160, 161]. 

Because all of these small calcium-binding proteins are water-soluble, they diffuse freely throughout the cytoplasm of the soma and processes of neurones in which they are expressed, often enabling visualization of even the smallest dendritic branches, thus yielding an appearance resembling Golgi impregnations. This property has enabled the classification of neurones by shape of the soma and neurites during development and at maturity, not as easily visualized by other neuronal markers [162]. Excellent reviews of this family of neuronal markers are provided by Baimbridge et al. [163] and Ulfig [163]. 

*Calmodulin* is the least specific of these markers and labels most neurones. The term often is applied as a general name for a group of calcium-binding proteins, expressed more in neurones than in glial cells. The three major members of this family, *calretinin*, *calbindin D28k* and *parvalbumin*, share many characteristics in common, but also differ from each other in their distributions in both the mature and immature brain, selective expression only in certain neurones and in the timing of onset of their expressions. The genetic locus of calretinin is on chromosome 16, whereas that of calbindin D28k is on chromosome 8 in humans, both genes being in close proximity to carbonic anhydrase isozyme genes [165]. Calbindin is so universally essential to life that it is even used by by plants in their metabolism [166]. 

**Figure 6:**
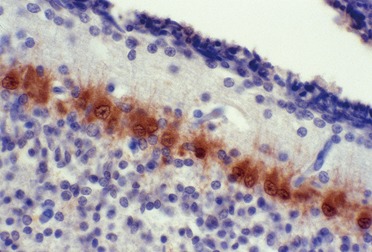
Figure 6. Calbindin D28k reactivity in cerebellar cortex of a 34-week fetus shows strong, selective reactivity in Purkinje cells and not in other neurones. The pattern is almost the opposite of NeuN (compare with Figure 1D). Their dendritic trunks extending into the molecular zone show early reactivity, but not the Golgi-like ramifications seen at term and post-natally (not shown). × 100, original magnification.

*Calretinin* was so named because of its strong reactivity in ganglion cells and photoreceptor neurones of the retina during development [167], first detected when an antibody against calbindin was found to cross-react with a slightly larger molecule in homogenated rat brain [168]. Calretinin is important throughout the nervous system, not just in the retina, because it specifically labels GABAergic inhibitory interneurones that arrive at the cerebral cortex and hippocampus by tangential, rather than radial, migrations. At least in the rodent, GABA is excitatory, rather than inhibitory because it mediates depolarization rather than hyper-polarization of the plasma membrane of immature neurones in fetal brain [169]. Calretinin also is reactive with other GABAergic neurones throughout the neuraxis, including those of the dentate and inferior olivary nuclei of the cerebellar system and basket cells of the cerebellar cortex. Purkinje cells are more weakly or non-reactive. Calbindin D28k, by contrast, shows selective intense reactivity in both immature and mature Purkinje neurones (Figure 6), labeling not only the soma, but the detailed tiny branches of the dendritic tree of mature cells within the molecular layer, reminiscent of Golgi impregnations [115]. This reactivity in Purkinje, Golgi, basket, stellate and deep cerebellar nuclear neurones appears as early as 12 – 14 weeks gestation [115, 170, 171]. Parvalbumin reactivity also is detected initially, but then regresses so that by 21 – 31 contrast, are not detected in the cerebellar cortex until 21 weeks and thereafter progressively increases with maturation [171]. In the retina, calbinden D28k is a specific marker for horizontal cells and correlates well with coexpression of choline acetyltransferase and acetylcholinesterase activities in the early postnatal rat [173]. Calretinin is strongly reactive in parasympathetic ganglion cells of the submucosal and myenteric plexi, and may be applied to bowel resections for possible Hirschsprung disease. 

**Figure 7:**
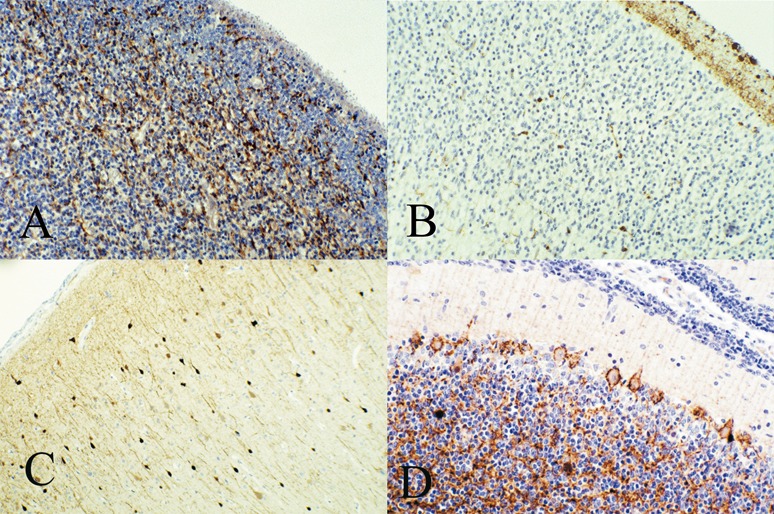
Figure 7. Calretinin reactivity in (A) many scattered cells of the ganglionic eminence adjacent to the lateral ventricle of a 15-week fetus. B: cerebral neocortex of a 21-week fetus showing strong reactivity in Cajal-Retzius neurones and fibers of the molecular zone and scattered reactive cells within the cortical plate comprising only 5% of total neurones. C: Cerebral neocortex of a term neonate, showing ~ 12% of total neurones are reactive with predominance in layer 2; both proximal axones and dendrites are labeled, demonstrating the bipolar shape and radial orientation of these neurones. D: In the cerebellar cortex of this infant, born at 42 weeks gestation who lived only 2 hours, after severe perinatal ischemia secondary to abruptio placentae, calretinin reactivity resembles that of synaptophysin, showing Purkinje cells surrounded by punctuate reactivity at synapses and reactivity in synaptic glomeruli of the internal granular layer. The overall reactivity in the cerebellum is less than expected for age and corresponds to about 35 weeks gestation. (A, B) × 200; (C, D) × 100, original magnifications.

Many pre-migratory neuroblasts are already labeled in the ganglionic eminence (Figure 7A); their tangential migratory pathway can be followed by the reactivity of both the cells and their neurites, and their distribution within the cortical plate can be shown [172]. Even before the first wave of radial migratory neuroblasts, at 6 week gestation, Cajal-Retzius neurones are strongly marked by calretinin; at mid-gestation ~ 5% of cortical neurones show reactivity (Figure 7B). By term, ~ 12% of cortical neurones are calretinin-positive (Figure 7C), the most important sub-population of the total GABAergic inhibitory interneurones that comprise about 20% of total cortical neurones. The distribution of calretinin-reactive neurones is predominantly in the layers of small granular cells that are mainly receptive, Layers 2 and 4, but there also are scattered marked neurones throughout all layers of the cortical plate. In mature cortex, calretinin-reactive neurones are seen to be bipolar with reactivity in both the axone and dendritic trunk (Figure 7C). In the cerebellar cortex, calretinin can exhibit an appearance reminiscent of synaptophysin, with localisation to synapses surrounding Purkinje neurones and in the synaptic glomeruli of the internal granular layer (Figure 7D). 

The subplate zone of the cerebral cortical plate is a transitory anatomical region of the fetal brain between 13 and 22 weeks gestation, located just beneath the neocortical plate. Functionally it is essential in the organisation of the cortical plate, synaptogenesis (subplate neurones receive the first cortical afferent connections) and in gyration [164]. Many of the subplate neuronal precursors arise in the ganglionic eminence and hence are strongly reactive with calretinin and calbindin D28k; from a practical perspective, these markers are excellent for studying maturation of the subplate [164, 173, 175, 178]. Calbindin is expressed after 25 weeks gestation [152], but calretinin is expressed earlier. After mid-gestation the subplate begins to thin and eventually disappear, in part by incorporation of some neurones into layer 6 of the cortex and in part by an accelerated apoptosis of neurones no longer needed [176, 177, 178, 179]. 

In the corpus striatum, a transitory patchy pattern of calbindin reactivity is seen in the neuropil from 16 weeks gestation to term [164, 178], similar to the patchy synaptogenesis demonstrated in the caudate nucleus and putamen [32, 33]. These patches of reactivity correspond to acetylchoinesterase (AChE)-rich zones [178] and to the striosomes of Graybiel [181]. Calretinin and calbindin D28k also exhibit a wide distribution and identify several neuronal types in the amygdala [164, 182], in the basal nucleus of Meynert in the 7^th^ month of gestation [161] and in the red nucleus [36], whereas parvalbumen-reactive neurones are seen in the hypothalamic tubero-mamillary nucleus of the adult [183], but this reactivity does not appear until the 7^th^ month of gestation [164]. Calbindin- and calretinin-reactive neurones are found in the rabbit superior colliculus [184] and the murine inferior colliculus during development [185]. 

#### S-100α protein 

S-100β protein is a classical marker of glial cells that has been used for decades, but the lesser known α form is a neuronal rather than a glial marker and is another calcium-binding protein [186]. Few data about its fetal expression are available. As with the enolases, S-100 proteins may be measured in serum and CSF as biomarkers of brain injury in the newborn [187] and after acute traumatic CNS injury [186]. The serum level is elevated in CSF in some [188], but not a reliable biomarker of brain dysfunction in most children with epilepsy [91]. High levels of circulating S-100 proteins in patients with severe traumatic head injuries indicate only acute, not chronic, injury because the serum half-life is only 2 hours [189]. 

#### Chromogranins 

The chromogranins are another family of water-soluble, calcium-binding proteins. They were discussed earlier because they are associated with synaptic vesicles. 

### Miscellaneous other neuronal markers 

#### Protein gene product-9.5 (PGP9.5) 

This is one of the more recent neuronal cytoplasmic markers and is general and non-specific for the type of neurone or its neurotransmitter. Most of the literature on this gene product relates to its expression in the enteric autonomic nervous system and its function as a tumour-suppressor gene for a variety of carcinomas [190, 191]. Though there are sparse data in the CNS, it does show reactivity with neurones of the brain. It is not expressed early in neuroepithelial cells, but during neuroblast migration and persists at neuronal maturity. Contrary to several reports of its value as a marker of mature myenteric plexus ganglion cells, other authors have found it to not be constant or useful for this purpose [192]. In my experience, PGP9.5 can be capricious and is not as reliable as many other neuronal markers in the immature brain. 

#### Markers of specific neurotransmitters, their enzymes of biosynthesis and degradation and neuropeptides 

There are many immunocytochemical methods now developed to show these specific products. They provide a specificity that compliments more universal neuronal markers such as NeuN, NSE and synaptophysin. A systematic discussion of these markers is beyond the scope of this present review. 

### Histochemical markers 

#### Luxol fast blue 

Myelination of axones is a final process in the development of neurones, hence stains that indicate myelin formation are late markers of maturation. The Klüver-Barrera method of myelin stain with luxol fast blue (LFB), counterstained with PAS or H&E, is useful for showing the myelination sequences of axones in white matter [193]. Weigert stain and its Loyez modification is another similar technique [194]. Even more precise staining can be achieved with modified gallocyanin stains (usually used as nuclear DNA and mitotic markers) and myelin basic protein immunoreactivity, and especially with transmission electron microscopy, but LFB remains the most readily available routine stain in most laboratories and is quite reliable. Marchi stain is traditional for demonstrating degenerating myelin. Atlases and tables detailing the sequence of myelination in the nervous system using luxol fast blue show the timing of myelination [195, 196, 197]. Myelination in tissue sections appears earlier than can be detected by MRI. 

#### Periodic acid-Schiff (PAS) reaction 

The PAS reaction is one of the earliest histochemical stains developed and is applicable to frozen and to paraffin sections. It stains glycogen in neurones, glial and ependymal cells and is digested by pre-incubation with diastase. It also stains polysaccharides that are not digested by diastase. Its application as a marker of neuronal maturation is based upon glycogen content of neurones, but there are so many variables that may affect glycogen content, including intravenous glucose infusions in the hours and days before surgical brain resection or before death, that it is difficult to use for this purpose. Furthermore, glycogen digestion continues after death, so it is not suitable for autopsy tissue. The amount of cytoplasm of a given neurone greatly influences the visibility of glycogen. Finally, glycogen is many times more abundant in glial cells, particularly astrocytes, in which it has a higher turnover rate, than in neurones [198, 199]. It is not recommended as a marker for defining neuronal maturation, despite its ease of application and familiarity by pathologists. The glycolytic enzyme NSE, by contrast, is not prone to the conditions and variables of cytoplasmic glycogen itself, hence is more reliable (see above). 

#### Acridine orange (AO) fluorochrome 

The family of aminoacridine molecules forms highly fluorescent bonds with nucleic acids. AO is the most useful amongst them for application to the nervous system. Because both the wavelengths of absorption of ultraviolet/blue light and of emission of visible light differ between AO-DNA and AO-RNA complexes, these two nucleic acids are perceived in the fluorescence microscope as different colours, AO-DNA appearing yellow-green and AO-RNA as brilliant orange-red [200, 201]. In neurones, the AO-RNA bonds are mainly with cytoplasmic ribosomes. AO may be demonstrated in either frozen or paraffin sections. It is a much more sensitive method for demonstrating ribosomal RNA than older methods of methyl-green-pyronin, hematoxylin or Nissl (cresylviolet) stains. 

Because the fluorescence fades with exposure to UV light, a permanent record can only be made by photography. A background diffuse pale orange fluorescence sometimes occurs in paraffin sections, but this artefact dissipates with exposure to UV light much sooner than the natural AO-RNA complexes, so the timing of photography is critical, awaiting the disappearance of artifactual background fluorescence but before the natural AO becomes quenched. Sections stained for AO are temporary mounts in water. After observing the AO fluorescence, the coverslip can be removed and the sections restained with H&E, or they may be air-dried and restained with AO at another time. AO quenches the autofluorescence of lipofuscin in neurones [202]. 

**Figure 8:**
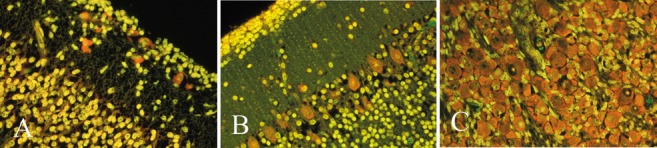
Figure 8. Acridine orange (AO) fluorochrome histochemistry. A: Cerebral cortex of an 18-week fetus shows intense orange-red fluorescence in Cajal-Retzius neurones of the molecular zone, but only faint orange colour in immature neurones of the cortical plate. The intense orange represents ribosomal concentration associated with neurotransmitter biosynthesis, hence serves as a maturational marker. B: Cerebellar cortex of a 38-week near-term infant showing strong AO-RNA fluorescence in Purkinje neurones and basket cells; the fluorescence in granule cells is faint because the rim of sparse cytoplasm is so thin. AO-DNA fluorescence of nuclei is perceived as yellow-green. C: Strong AO-RNA fluorescence in the large primary sensory neurones of a dorsal root ganglion of a 15-week fetus. (A, B, C) × 250, original magnifications.

In maturing neurones, a proliferation of ribosomes and of granular endoplasmic reticulum is associated with the initiation of neurotransmitter synthesis in the neurone, and the AO-RNA fluorescence is a marker of this stage of maturation [201]. All functional neurones show orange-red AO-RNA fluorescence, but the intensity is greatest in large cells with abundant cytoplasm, such as motor neurones and Purkinje cells (Figure 8B). In the neocortical plate at mid-gestation, Cajal-Retzius cells of the molecular zone are intensely fluorescent, whereas the maturing neuroblasts of the cortex exhibit only a pale orange fluorescence (Figure 8A). Migratory neuroblasts along radial glial fibers the subcortical white matter sometimes show pale orange cytoplasmic fluorescence before arriving at the cortical plate. Neurones of the cerebellar dentate nucleus and inferior olivary nucleus exhibit early fluorescence from ~ 13 – 15 weeks gestation. Motor neurones of cranial nerves and ventral horns of the spinal cord, as well as primary sensory neurones of cranial nerve and spinal dorsal root ganglia (Figure 8C) also show early fluorescence. AO also is useful in demonstrating maturing ganglion cells in the myenteric and submucosal plexi of the intestine in infants [203]. 

#### Mitochondrial respiratory chain “oxidative” enzymes 

The three respiratory chain enzymes that can be demonstrated histochemically in muscle biopsies also can be applied to frozen sections of brain tissue, but not to formalin-fixed paraffin sections: nicotinamide dinucleotide-tetrazolium reductase (NADH-TR; Complex I); succinate dehydrogenase (SDH; Complex II); and cytochrome-c-oxidase (COX; Complex IV). 

Mitochondrial enzymes can indicate a stage of maturation of immature neurones when they require a larger energy metabolism to maintain a resting membrane potential and to for the synthesis of neurotransmitters. 

**Figure 9:**
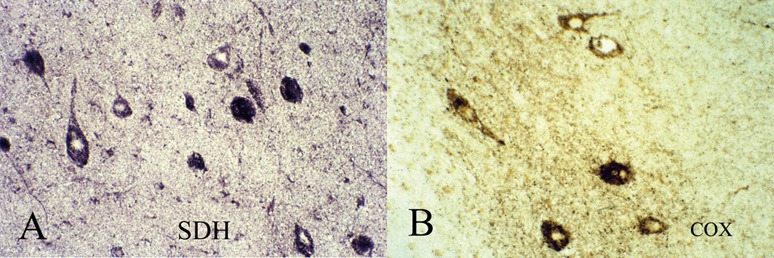
Figure 9. A: Succinate dehydrogenase (SDH; respiratory complex II) and (B) cytochrome-c-oxidase (COX; respiratory complex IV) histochemical activities of frozen sections of right temporal neocortical resection of a 2-month-old male infant with severe right hemimegalencephaly and refractory epileptic activity arising in this focus. Scattered neurones exhibit intense mitochondrial oxidative activity and probably were continuously discharging. Neurones with normal activity in the background are difficult to identify because the neuropil shows almost equal low-grade activity. The tissue architecture and orientation of neurones also is severely altered. Epileptic foci in cortical tubers and in focal cortical dysplasias, Types 1 and 2, exhibit a similar pattern of scattered neurones with intense mitochondrial activity. Electron microscopy demonstrated increased numbers of mitochondria with normal-appearing cristae in such neurones (not shown). Reproduced with permission from reference [204]. (A, B) × 200, original magnifications.

Such enzyme histochemistry has been successfully applied to intractable epileptic foci in surgical resections of cortex in children and shows scattered neurones with greater than expected intensity of activity that are interpreted as “hypermetabolic” neurones that are frequently or continuously discharging in the epileptic focus; in tuberous sclerosis and hemimegalencephaly some of the dysplastic neurones also show increased mitochondrial enzymatic activities (Figure 9A,B) [204]. The normal control activity at various gestational ages is currently under investigation in our laboratory, but one question not yet resolved is whether there is post-mortem loss of activity, rendering the technique reliable only when applied to surgical resections but not to fetal autopsies. A disadvantage is that it requires frozen sections and planning in advance, at the time of brain removal at fetal autopsy. This limitation makes it impractical at times. However, immunocytochemical antibodies against mitochondrial respiratory chain enzymes are now becoming available that are suitable for formalin-fixed, paraffin-embedded sections, though it remains uncertain at this time whether they are as precise and reliable as the traditional histochemical activities. 

#### Argentophilic impregnations 

**Figure 10:**
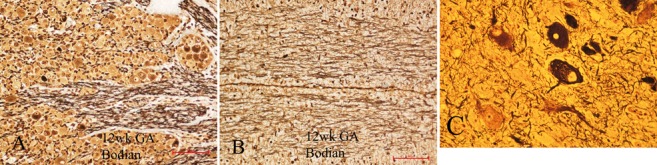
Figure 10. A, B: Bodian silver impregnations in a 12-week fetus, showing (A) nerve fibres emerging from a lumbar dorsal root ganglion, and (B) coarse axones of the dorsal columns on either side of the dorsal median septum of lumbar spinal cord, sectioned in the horizontal plane. C: Bielchowsky technique demonstrates dense aggregation of somatic neurofilaments (neurofibrillary tangles) in several neurones within a cortical tuber of a 3-year-old boy; another neurone appears normal without this condensation. Similar neuronal findings are observed in late gestational fetal brains with tuberous sclerosis. (C) × 400, original magnification.

Silver deposits on neurofilaments to demonstrate large axones have been used for more than a century, with variations on a basic technique: Bielschowsky; Bodian (protargol); Sevier-Munger; Palmgren and others [194, 205]. Axones are impregnated in developing tracts and in nerve roots as early as 12 weeks (Figure 10A, B). They normally do not impregnate neurofilaments within the somatic cytoplasm at any age. They are useful, however, for showing neurofibrillar aggregates within neuronal cell bodies in pathological conditions, particularly in dysplastic neurones in focal cortical dysplasia Type 2, and in hamartomata with cytological dysgenesis as in tuberous sclerosis (Figure 10C) and hemimegalencephaly. 

Though neither Bodian nor Bielschowsky methods provide the precision in demonstrating fine processes as is demonstrated by Golgi impregnations, these cruder techniques are more practical for most neuropathology laboratories and do yield useful information. In my experience, silver Golgi is a more reliable technique for immature brain tissue than is Golgi-Cox mercury (corrosive sublimate). These impregnations can serve as markers of maturation by their demonstration of axones and, in the case of silver Golgi technique, of the dendritic tree and spines that denote the transition from neuroblast to mature neurone. The fine detail provided by Golgi impregnations is unique at the light microscopic level, and was used to advantage in describing developing brain more than a century ago by Ramón y Cajal [206] and also in more contemporary studies, such as those of Marín-Padilla [207]. 

Special silver impregnation techniques are designed for degenerating axones [194, 205, 208], but these are not relevant to the study of young developing brains. They might be useful to demonstrate the normal postnatal loss of rubrospinal axones, for example, or the loss of axones from neurones of the thick early fetal septum as it undergoes regressive changes to become a thin membranous septum pellucidum. 

## Discussion 

A fundamental principle of neuroembryology is that genetically programmed development is precise both in morphogenesis and in the timing of initiation and progression of each of the several developmental processes over an extended period of ontogenesis. Neuronal markers from a developmental perspective are all about timing. Onset of expression of these markers is an aspect of brain development too infrequently addressed. 

Genetic mutations may alter the timing and sequence of synaptogenesis in fetal holoprosencephaly as an example: either delayed or precocious maturation of neocortical synapse formation in this prototype malformation of genetic programming [209]. Precociousness is seen both at excitatory axodendritic synapses (molecular zone) and inhibitory axosomatic synapses (within the cortical plate surrounding neurones), as well as earlier than expected expression of NeuN [209]. Mutations that inactivate one copy of the gene *SYNGAP1* causes intellectual disability, autism and epilepsy, and results in precocious synaptogenesis during an early window of development [42]. Maturational delay in myelination is seen in many inborn metabolic diseases, documented both in MRI during life and neuropathologically in infants who do not survive. Mice with homozygous mutations in MAP-1 exhibit major delays or arrests in CNS development including cytological maturation [210]. Delayed maturation can affect not only the central, but also the peripheral, nervous system. We have recently found delayed maturation of NeuN and other neuronal protein markers in ganglion cells, as well as sparse synaptophysin reactivity in the myenteric plexi in a series of small bowel segmental resections and at autopsy in preterm infants with intestinal dysmotility syndrome, which explains poor peristalsis [Yu W, Pinto-Rojas A, Sarnat HB, in preparation]. 

Mid-gestation is an important transitional time in cerebral corticogenesis. Three important changes occur at ~ 20 weeks: 1) regression, but not yet disappearance, of the subplate zone [163]; 2) change from radial micro-columnar architecture of the fetal cortical plate to superimpose the histological layering that characterizes the mature cerebral cortex; synaptic layers also shift from radial to horizontal, as shown by synaptophysin [211]; 3) initiation of sulcation and gyration of the cortex; fissures develop much earlier but are of different character and ontogenesis [212]. Though abnormal convolutions (pachygyria; polymicrogyria) and lack of convolutions (lissencephaly) are associated with neuroblast migratory disorders and abnormal cortical architecture in many specific genetic disorders of brain development, more than 90% of both radial and tangential migration in the telencephalon is completed before gyration even begins [213, 214]. 

Despite the shift from radial columnar architecture of the cortical plate at mid-gestation, lamina-specific markers demonstrate the primordial horizontal architecture before it is evident histologically [215, 216]. The tangentially migratory GABAergic inter-neurones and Cajal-Retzius neurones are important in the reorganization of the cortical plate [217, 218, 219]. 

Immunocytochemical markers of neuronal maturation are useful in showing normal and identifying abnormal initial distribution of these neurones. An example is the displacement of NeuN-reactive large neurones in the superficial, rather than the deep, part of the cortical plate in holoprosencephaly [209], suggesting that the inside-out pattern of distribution of the waves of radial migration did not occur because neurones in the deep layers normally show NeuN expression sooner than the superficial layers, as illustrated in Figure 1A, B; this inversion also is seen in the abnormal nodular architecture of some cortical dysgenesis patterns (Figure 1F). Another example is foetal hydrocephalus at 32 – 34 weeks gestation, in which distinct alterations are demonstrated in the distribution of calbindin D28k and parvalbumin in the subplate of the occipital lobe, compared with age-matched controls, though the expected pattern of distribution within the cortical plate itself remains preserved [220]. 

In malformations of the brain, timing of expression of neuronal proteins also usually are aberrant in those involving cytologically abnormal neurones, as in tuberous sclerosis, hemimegalencephaly and focal cortical dysplasia Type 2. Documentation of normal patterns is essential to enable interpretation of abnormal neuronal maturation in malformations, complimenting abnormal tissue architecture. For example, the *balloon cells* seen in focal cortical dysplasia Type 2B in resected temporal lobe of epileptic patients show diminished MAP2 expression [136]. Markers of glial and ependymal cell maturation will be addressed later in a separate review. 

Traditionally malformations of the brain are generally defined by abnormalities in tissue architecture and whether the neural cells themselves are cytologically normal or abnormal. Nodules of altered cells with abnormal growth, differentiation and lineage within abnormal tissue architecture are known as *hamartoma*. Examples of cytological dysgenesis are tuberous sclerosis [137, 221, 222], hemimegalencephaly [138, 223], Type 2 focal cortical dysplasias [135, 224] and dysplastic gangliocytoma of the cerebellum (Lhermitte-Duclos/Cowden disease) [225]. Abnormal inhibitory circuits in cortical tubers of tuberous sclerosis, associated with refractory epilepsy, are accompanied by aberrant expression of parvalbumin and calbindin D28k in the dysplastic cortex [226]. The density of calretinin-reactive neurones is reduced in focal cortical dysplasias by 70% in Type 1 and by 50% in Type 2 [227]. Since calcium-binding protein mainly label GABAergic inhibitory interneurones in the cortex, a decreased number of these cells might at least partially explain why such cortex is epileptogenic. In hippocampal sclerosis, by contrast, calretinin- and calbindin-reactive neurones are well preserved [228, 229], despite extensive loss of pyramidal and granular neurones that do not express calcium-binding proteins. Calretinin-reactive neurones may even increase after febrile seizures [230]. 

The cellular markers here discussed not only demonstrate neuronal maturation, but also are more sensitive than H&E and other histological stains in detecting ischaemic neuronal insults. Hypoxic/ischaemic encephalopathy is perhaps the most common neuropathological condition encountered in neonatal autopsies. Distinctive alterations are seen with synaptophysin in cerebral infarcts, laminar necrosis of the cortex and other ischaemic lesions [31]. MAP proteins also are sensitive markers of ischaemic cellular changes in neonatal mice and humans [231, 232, 233]. AO fluorochrome exhibits a centrifugal (chromatolytic) distribution of ribosomal RNA in ischaemic neonatal motor neurones in humans and rodents [234, 235], by contrast with early degenerating motor neurones in infantile spinal muscular atrophy (Werdnig-Hoffmann disease) in which the motor neuronal cytoplasmic distribution of AO-RNA complexes tend to be centripetal (perinuclear) [202]. 

In conclusion, modern neuropathological examination of fetal and neonatal brain and of malformations of the nervous system at any age require documentation of cytological maturation as well as the arrangement of cells as tissue architecture. Of the many markers of neuronal maturation commercially available, the single most useful one for the practicing diagnostic neuropathologist is synaptophysin, which also is highly resistant to post-mortem degradation. The next most useful for fetal brain are NeuN and calretinin. These antibodies are already used in most neuropathology laboratories for other purposes. 

## Acknowledgment 

I am grateful to Dr. Weiming Yu, pediatric pathologist at Alberta Children’s Hospital, for reading the manuscript and offering helpful suggestions. I also thank Gaston Guenette and Patricia McInnis of the Histopathology Laboratory at Alberta Children’s Hospital, and Vivian King and her staff in the Immunopathology Laboratory of Calgary Laboratory Services, for their meticulous technical preparation of fetal brain tissue sections. 

Presented as the Gordon Mathieson Invited Member Lecture at the 50^th^ Anniversary Meeting of the Canadian Association of Neuropathologists, Toronto, Ontario, October 13 – 16, 2010.

## Conflict of interest 

The author has no conflicts of interest or financial disclosures to declare. The surgical resection and post-mortem neuropathological examinations here reported fulfill the ethical guidelines and Conjoint Health Research Ethics Committee at the University of Calgary and Alberta Health Services. 

**Table 1:** Immunocytochemical markers of neuronal maturation.

		Post-mortem	Quality**
Antibody	Onset*	Reliability**	After 10 years
Nucleoproteins			
Neuronal nuclear antigen (NeuN)	late	1 day	preserved
Synaptic vesicle proteins			
Synaptophysin	late	5 days	preserved
Chromogranin-A (CgrA)	late	2 days	faded 50%
Enolases			
Neurone-specific enolase (NSE)	intermediate/late	3 days	faded 70%
Cytoskeletal proteins			
Microtubule-associated protein-2 (MAP2)	early	3 days	preserved
Class III β-tubulin (TUJ1)	early	3 days	preserved
Doublecortin (DCX)	early	3 days	preserved
Tau (non-phosphorylated)***	early	3 days	faded 70%
Neurofilament proteins (NFP)	early/intermediate	3 days	faded 50%
α-Internexin	early	3 days	?
Vimentin***	early	5 days	preserved
Calcium-binding soluble proteins			
Calretinin	early	3 days	faded 30%
Calbindin D28k	intermediate	3 days	faded 30%
Parvalbumin	intermediate	3 days	?
Transcription product			
Protein gene product 9.5 (PGP9.5)	intermediate	3 days	preserved

*, **, ***See Table 2.

**Table 2:** Histochemical markers of neuronal maturation.

		Post-mortem	Quality
Product	Onset*	Reliability**	After 10 years
Luxol fast blue	late	5 days	preserved
Periodic acid-Schiff (PAS) reaction*****	late	12 – 24 hours	preserved
Acridine orange (AO) fluorochrome	intermediate/late	5 days	fades 5 minutes
Mitochondrial oxidative enzymes****	late	30 minutes	preserved
Bielschowsky or Bodian silver impregnation	late	5 days	preserved

*Late onset is defined as expression only after the neurone initiates transmitter synthesis and forms synapses. Intermediate onset is defined by expression in neuroblasts during migration, before transmitter synthesis and synaptogenesis, but not as progenitor cells. Early onset is defined by expression as progenitor neuroepithelial cells with early neuronal lineage, before migration initiated. **Post-mortem reliability indicates the period between fetal death and fixation of tissue. Some stillborn fetuses may have died 1 – 3 days before delivery and autolysis in those cases would have proceeded rapidly because the tissues were at the mother’s body temperature. Quality after 10 years refers to the preservation of reactivity in archived microscope slides prepared 10 years earlier. ***Tau and vimentin are the only proteins in this list that is expressed early and then disappear from immunocytochemical demonstration in intermediate stages of neuronal differentiation. ****Frozen, unfixed sections required; more suitable for surgical than autopsy tissue. *****Glycogen digestion within cells, including neurones, continues post-mortem.
